# Efficacy and safety of esketamine for pediatric gastrointestinal endoscopy: a meta-analysis and trial sequential analysis

**DOI:** 10.3389/fphar.2024.1379101

**Published:** 2024-04-25

**Authors:** Yunfeng Yu, Juan Deng, Keke Tong, Yuman Yin, Rong Yu, Chuanchuan Tan

**Affiliations:** ^1^ The First Hospital of Hunan University of Chinese Medicine, Changsha, Huanan, China; ^2^ The Third Hospital of Changsha, Changsha, Huanan, China; ^3^ College of Chinese Medicine, Hunan University of Chinese Medicine, Changsha, Hunan, China

**Keywords:** esketamine, low dose, gastrointestinal endoscopy, children, meta-analysis, trial sequential analysis

## Abstract

**Objective:**

The role of esketamine in pediatric gastrointestinal endoscopy is still unclear. This study aims to evaluate the efficacy and safety of esketamine for pediatric gastrointestinal endoscopy.

**Methods:**

Clinical trials of esketamine for pediatric gastrointestinal endoscopy were searched in eight common databases, up to October 2023. These clinical trials were included in the meta-analysis and trial sequential analysis (TSA). The risk ratio (RR) and weighted mean difference (WMD) were used as the effect sizes for dichotomous variables and continuity variables, respectively. When the heterogeneity test showed I^2^ < 50%, the fixed effects model was used for the meta-analysis and TSA; Otherwise, the random effects model was used for them.

**Results:**

In terms of efficacy endpoints, the meta-analysis showed that compared with placebo or blank, esketamine significantly decreased recovery time by 2.34 min (WMD −2.34; 95% Confidence interval [CI] −3.65, −1.02; *p* = 0.0005) and propofol consumption by 0.70 mg/kg (WMD −0.70; 95% CI −0.98, −0.43; *p* < 0.00001), and increased mean heart rate by 4.77 beats/min (WMD 4.77; 95% CI 2.67, 6.87; *p* < 0.00001) and mean arterial pressure by 3.10 mmHg (WMD 3.10; 95% CI 1.52, 4.67; *p* = 0.0001), while induction time and mean blood oxygen remained comparable. TSA indicated conclusive evidence for these benefits. In terms of safety endpoints, the meta-analysis revealed that esketamine significantly reduced involuntary movements by 59% (RR 0.41; 95% CI 0.22, 0.76; *p* = 0.005) and choking by 51% (RR 0.49; 95% CI 0.26, 0.92; *p* = 0.03), while significantly increasing dizziness by 98% (RR 1.98; 95% CI 1.11, 3.56; *p* = 0.02) and there were no significant differences in total adverse events, respiratory depression, and vomiting. TSA demonstrated conclusive evidence for involuntary movements and dizziness. Low-dose analysis showed that esketamine at ≤0.3 mg/kg significantly reduced recovery time, propofol consumption and involuntary movements, and significantly increasing mean heart rate, with no increase in dizziness. The Begg’s test (*p* = 0.327) and the Egger’s test (*p* = 0.413) indicated no significant publication bias, yet the funnel plot suggested potential publication bias.

**Conclusion:**

Esketamine is an effective adjuvant anesthesia for children undergoing gastrointestinal endoscopy. However, the general dose of esketamine may increase the risk of dizziness, which can be avoided by administering a low dose (≤0.3 mg/kg).

## 1 Introduction

Gastrointestinal endoscopy is the gold standard for diagnosing digestive diseases, which is greatly significant for early detection and prevention of gastrointestinal diseases (Wallace et al., 2017). However, due to its invasiveness and discomfort, patients may experience adverse events such as nausea, vomiting, abdominal pain, bloating, anxiety and fear when undergoing gastrointestinal endoscopy (Zheng et al., 2018). In order to minimize patient discomfort and enhance the success rate of endoscopy, the “Guidelines for sedation and anesthesia in GI endoscopy” recommend the use of sedative-anesthetic drugs in conjunction with endoscopy (Early et al., 2018). It has been reported that the sedation rate for gastrointestinal endoscopy in China is about 50% (Zhou et al., 2021), while in the United States and Europe, the sedation rate is up to more than 90% (Cohen et al., 2006; Riphaus et al., 2013). In view of the fact that it is difficult for children to autonomous cooperate to complete the examination, they need to receive sedation and anesthesia to ensure the success of the examination (Zhang et al., 2022). Due to its fast onset, good efficacy and rapid metabolism, propofol is widely used for pediatric endoscopic and imaging examinations (Rutman, 2009; Kiriyama et al., 2014). Although propofol can shorten the anesthetic induction time and recovery time for gastrointestinal endoscopy compared to conventional sedatives (Zhang et al., 2018), there is still a concern about its potential adverse events such as respiratory depression, bradycardia and hypotension (Feng et al., 2022).

Esketamine is an isomer of ketamine and an N-Methyl-D-Aspartate (NMDA) receptor antagonist (Zhan et al., 2022). Compared to conventional racemic ketamine, it has better sedative and analgesic effects as well as safety (Pfenninger et al., 2002). Although the European Medicines Agency (EMA) and National Medical Products Administration (NMPA) have approved the use of esketamine for general anesthesia, the Food and Drug Administration (FDA) in the United States only approves its use for treating depression. Recent meta-analyses have demonstrated that coadministration of esketamine with propofol significantly reduces recovery time, propofol consumption and associated complications in Chinese adults undergoing gastrointestinal colonoscopy (Lian et al., 2023). However, due to the lack of relevant large-sample and multicenter clinical evidence, the benefits and risks of esketamine for the pediatric gastrointestinal endoscopy remain unclear. Therefore, in this study, we use the meta-analysis and trial sequential analysis (TSA) to assess the efficacy and safety of esketamine for pediatric gastrointestinal endoscopy. It aims to provide evidence-based evidence for the use of esketamine in pediatric gastrointestinal endoscopy.

## 2 Methodology

This study strictly followed the Preferred Reporting Items for Systematic reviews and Meta-Analyses (PRISMA) (Page et al., 2021) and was registered in Prospero CRD42024530125, www.crd.york.ac.uk/prospero/display_record.php?RecordID=530125.

### 2.1 Literature search

A search method combining subject terms and extra terms was employed to retrieve relevant literature. The subject terms used were “esketamine” and “gastrointestinal endoscopy.” The extra terms were obtained from the Mesh and Sinomed. Five English databases (Embase, PubMed, the Cochrane Library, Web of Science, Scopus) and three Chinese databases (China National Knowledge Infrastructure, WanFang, VIP) were searched for literature on the use of esketamine in the pediatric gastrointestinal endoscopy, with a time cutoff of October 2023. There were no language or other restrictions.

### 2.2 Inclusion and exclusion criteria

Inclusion criteria: 1) Randomized controlled trials (RCTs) in study design; 2) Children undergoing gastrointestinal endoscopy as the study population; 3) Children in the control group received anesthesia with propofol, while children in the experimental group received anesthesia with propofol and esketamine; 4) Efficacy endpoints included recovery time, induction time, mean heart rate, mean arterial pressure, mean blood oxygen, and propofol consumption. Recovery time referred to the time from the end of the gastrointestinal endoscopy to the patient becoming conscious. Induction time denoted the time from the start of propofol infusion to the patient entering general anesthesia. Both recovery time and induction time were recorded by an anesthesiologist or assistant. The mean heart rate, mean arterial pressure, and mean blood oxygen referred to the average values of the patient’s heart rate, arterial pressure, and blood oxygen recorded by the monitor during the gastrointestinal endoscopy. Propofol consumption was calculated as: (the total amount of propofol in the syringe before anesthesia—the remaining amount of propofol in the syringe after the examination)/the weight of the patient. The propofol consumption was recorded and calculated by an anesthesiologist or assistant. Safety endpoints included total adverse events, involuntary movements, choking, respiratory depression, vomiting and dizziness. They referred to adverse events that occurred between the start of anesthesia and the patient’s departure from the care unit, which were recorded by an anesthesiologist or assistant.

Exclusion criteria: 1) Data were published repeatedly; 2) Data were incomplete; 3) Data were not available.

### 2.3 Literature screening, data analysis, and bias risk

Firstly, all the literature was imported into Endnote X9 for screening, and duplicate and irrelevant literature was excluded based on the predefined inclusion and exclusion criteria. The remaining literature met the requirements of this study and was included. Secondly, Excel 2010 was used to record the basic characteristics and research data of each included literature. The basic characteristics included author name, publication year, sample size, intervention, examination type, male ratio, average age, average body mass index (BMI), and American Society of Anesthesiologists Physical Status Classification System (ASA) I ratio. Research data referred to any indicators related to predefined efficacy and safety endpoints. Thirdly, the Cochrane Risk of Bias Tool, carried by RevMan5.3, was used to assess the risk of bias for each included study. Items assessed included random sequence generation, allocation concealment, blinding of participants and personnel, blinding of outcome assessment, incomplete outcome data, selective reporting, and other bias. According to the evaluation criteria defined in the Cochrane Handbook for Systematic Reviews of Interventions, the bias risk for each study was assessed as low risk, high risk, or unclear risk (Higgins et al., 2019). These tasks were independently performed by Yunfeng Yu and Juan Deng, with any disagreements resolved by Chuanchuan Tan.

### 2.4 Statistical analysis

Revman5.3 was used for the meta-analysis. The risk ratio (RR) served as the effect size for dichotomous variables. And weighted mean difference (WMD) was used as the effect size for continuous variables when included studies use the same measurement method; Otherwise, standardized mean difference (SMD) was used as the effect size for continuous variables. Heterogeneity was assessed using the I^2^ test, and a fixed-effects model was used for analysis when I^2^ < 50%. In the event of methodological heterogeneity without apparent statistical heterogeneity, a pre-planned sensitivity analysis and subgroup analysis were intended to be conducted. They would explore the potential impact of methodological and clinical heterogeneity on outcomes by analyzing study design, participant characteristics, dose of esketamine, or other relevant factors to ensure the robustness of the meta-analysis results. A random-effects model was used for analysis when I^2^ ≥ 50%. In order to investigate and identify the sources of heterogeneity in the meta-analysis when the I^2^ ≥ 50%, subgroup analysis based on participant characteristics and dose of esketamine, as well as sensitivity analysis based on study design and leave-one-out method would be performed. These steps helped to better understand the sources of heterogeneity among studies and ensure the robustness of these findings. The statistical significance of the meta-analysis was *p* < 0.05.

Trial Sequential Analysis 0.9.5.10 Beta was used for trial sequential analysis (TSA). TSA was a statistical method that combines aspects of traditional meta-analysis with the principles of sequential analysis to evaluate the robustness of the findings and determine whether a conclusive result had been reached. It aimed to control the risks of random errors and repeated significance testing by calculating the required information size and monitoring the Z-curve to ascertain when the cumulative evidence crossed the trial sequential monitoring boundaries. In this analysis, type I errors and type II errors were set to 0.05 and 0.20, respectively. The RR reduction or mean difference was calculated based on the meta-analysis results, and the effect model was consistent with the meta-analysis. When the Z-curve crossed the trial sequential monitoring boundaries, the meta-analysis results observed in the current information were conclusive.

The funnel plot, Begg’s test, and Egger’s test were used to comprehensively evaluate publication bias. Firstly, a funnel plot was generated using Revman5.3, allowing for a visual examination of study results’ distribution and symmetry. Asymmetric scatter distribution on both sides of the funnel plot indicated potential publication bias. Then, we conducted Begg’s test and Egger’s test using Stata15.0 to provide statistical assessments of publication bias. The *p*-value of Begg’s test and Egger’s test was calculated using WMD and seWMD, with a *p*-value ≤ 0.1 indicated potential publication bias.

## 3 Results

### 3.1 Literature screening

**FIGURE 1:**
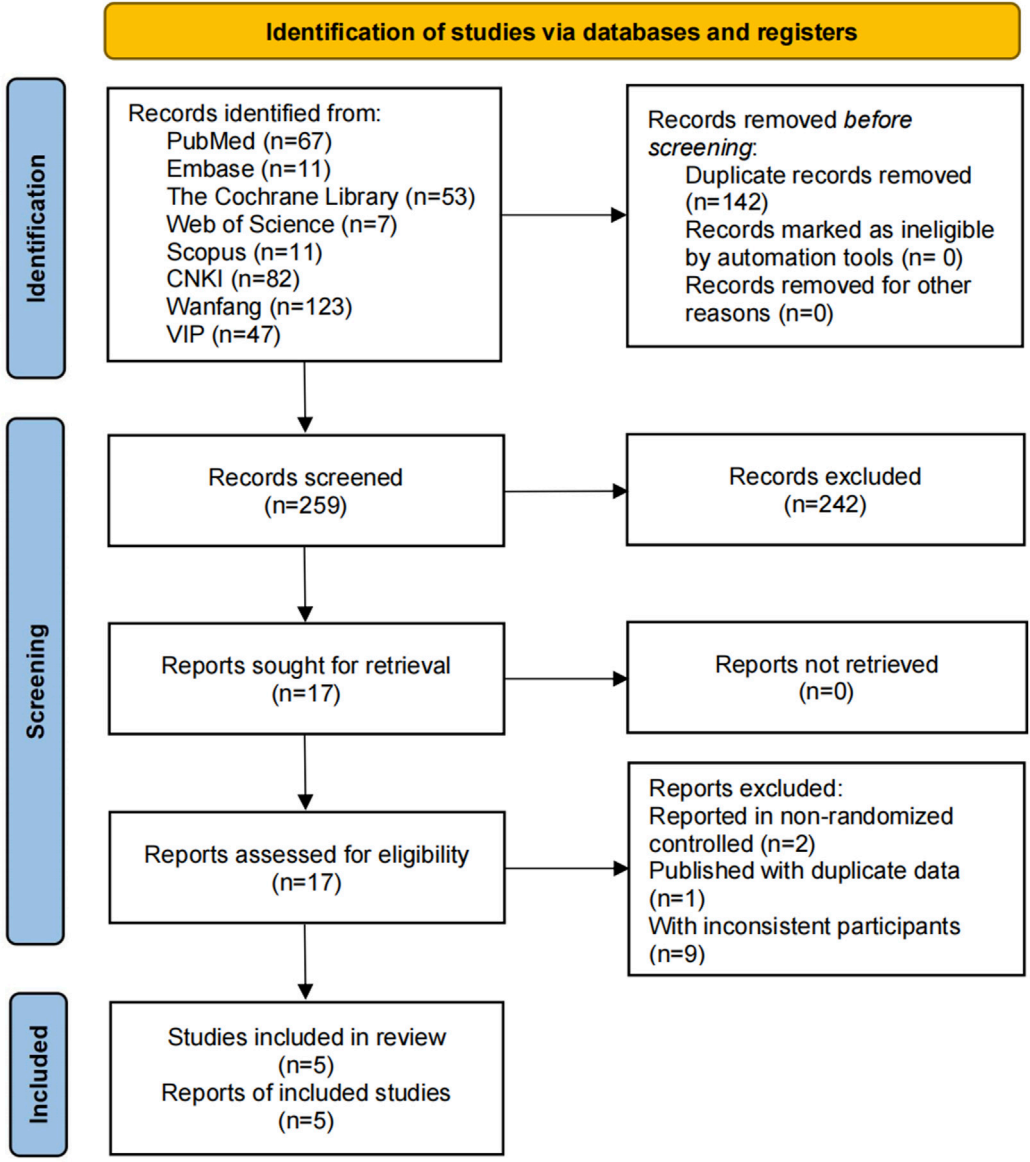
Literature screening flowchart.

A total of 401 relevant studies were identified from the databases. During the screening process, 142 studies were excluded due to duplication, and 242 studies were excluded during the review of titles and abstracts. Subsequently, we reviewed 17 full texts and excluded 12 studies from them. Among them, two studies were excluded due to non-randomized controlled design, one study was excluded due to duplicate data, and nine studies were excluded due to the inclusion of adults. Finally, we included five studies (Wang et al., 2022; Zhang et al., 2023; Ding et al., 2023; Li et al., 2023; Zheng et al., 2023). As shown in Figure 1.

### 3.2 Basic characteristics of included studies

A total of five clinical studies involving 549 patients were included. Among them, 304 patients received anesthesia with esketamine combined with propofol, while 245 patients received anesthesia with propofol alone. All trials were conducted in China, and the publication years ranged from 2022 to 2023. The author name, publication year, patient number, intervention, examination type, male ratio, average age, BMI, and ASA I ratio for each study are shown in Table 1.

**TABLE 1:** Basic characteristics of the included studies.

Author name	Patient number	Intervention	Examination type	Male (%)	Age (years)	BMI (kg·m^−2^)	ASA Ⅰ (%)
Ding et al. (2023)	37	0.3 mg/kg Esketamine	Colonoscopy	51.3	6.3	17.88	56.8
		2.0 mg/kg Propofol					
	37	0.9% NaCl	Colonoscopy	45.9	6.6	18.01	67.6
		2.0 mg/kg Esketamine					
Li et al. (2023)	38	0.15 mg/kg Esketamine	Gastroscopy	57.9	6.9	22.37	65.8
		2.5 mg/kg Propofol					
	38	2.5 mg/kg Propofol	Gastroscopy	52.6	6.8	22.56	63.2
Wang et al. (2022)	29	0.3 mg/kg Esketamine	Gastrointestinal endoscopy	58.6	9.9	18.13	—
		3.0 mg/kg Propofol					
	30	0.5 mg/kg Esketamine	Gastrointestinal endoscopy	56.7	8.9	17.4	—
		3.0 mg/kg Propofol					
	30	0.7 mg/kg Esketamine	Gastrointestinal endoscopy	53.3	9.5	18.37	—
		3.0 mg/kg Propofol					
	30	0.9% NaCl	Gastrointestinal endoscopy	43.3	9.4	17.1	—
		3.0 mg/kg Propofol					
Zheng et al. (2023)	100	0.5 mg/kg Esketamine	Gastroscopy	54.0	8.2	18.87	59.0
		2.5 mg/kg Propofol					
	100	3.0 mg/kg Propofol	Gastroscopy	53.0	8.2	18.78	58.0
Zhang et al. (2023a)	40	0.2 mg/kg Esketamine	Gastroscopy	57.5	8.7	—	87.5
		1.5–2.0 mg/kg Propofol					
	40	0.9% NaCl	Gastroscopy	60.0	8.5	—	87.5
		1.5–2.0 mg/kg Propofol					

BMI refers to body mass index; ASA I refers to the American Society of Anesthesiologists Physical Status Classification System as Class I.

### 3.3 Bias risk assessment

**FIGURE 2:**
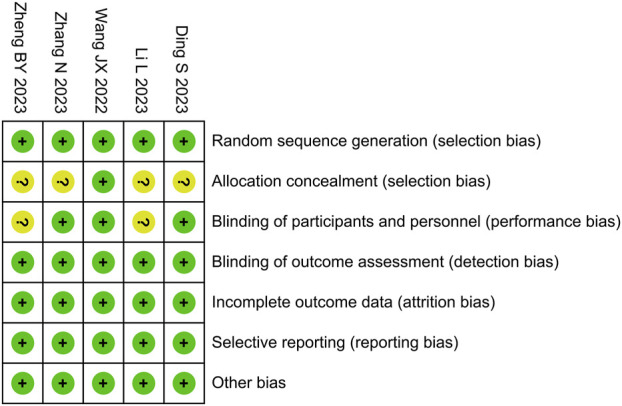
Risk of bias summary.

We used the Cochrane Risk of Bias Tool to evaluate the risk of bias in the included studies, as shown in Figure 2. The risk of bias for allocation concealment was unclear in four studies, the risk of bias for blinding of participants was unclear in two studies, and the risk of bias was low in the remaining areas. Among the five studies included, the overall risk of bias was assessed as low in three studies (Wang et al., 2022; Zhang et al., 2023; Ding et al., 2023) and as high in two studies (Li et al., 2023; Zheng et al., 2023).

### 3.4 Meta-analysis and trial sequential analysis

#### 3.4.1 Time endpoints

##### 3.4.1.1 Recovery time

**FIGURE 3:**
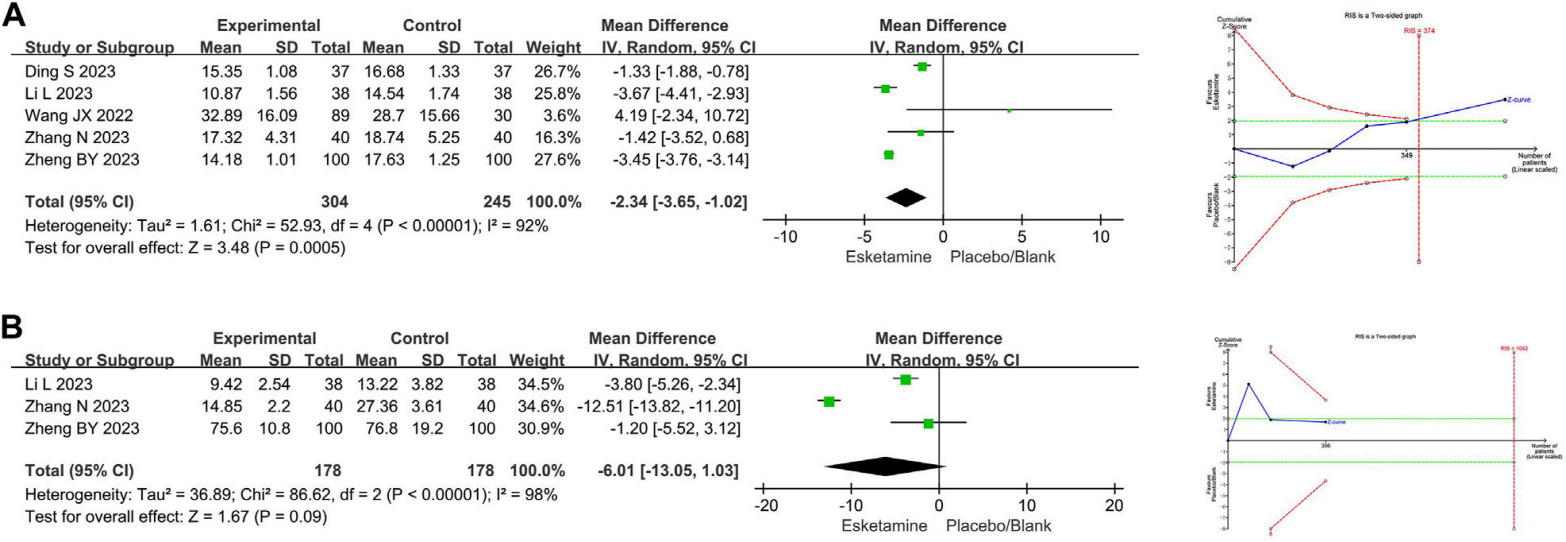
Meta-analysis and trial sequential analysis results for time endpoints of esketamine in pediatric gastrointestinal endoscopy. **(A)** Recovery time; **(B)** Induction time. MD refers to the weighted mean difference.

Five RCTs compared recovery time between the esketamine combination group and the propofol group, and they included 549 children undergoing gastrointestinal endoscopy. Among them, three studies had a low risk of bias (Wang et al., 2022; Zhang et al., 2023; Ding et al., 2023), and two studies had a high risk of bias due to lack of blinding of participants and allocation concealment (Li et al., 2023; Zheng et al., 2023). The meta-analysis showed that compared to the propofol group, the esketamine combination group significantly reduced the recovery time by 2.34 min (WMD −2.34; 95% confidence interval [CI] −3.65, −1.02; *p* = 0.0005; I^2^ = 92%). TSA indicated that the Z-curve of the recovery time crossed the boundary in the fifth study, suggesting it was conclusive. As shown in Figure 3A.

##### 3.4.1.2 Induction time

Three RCTs compared induction time between the esketamine combination group and the propofol group, and they included 356 children undergoing gastrointestinal endoscopy. Among them, one study had a low risk of bias (Zhang et al., 2023), and two studies had a high risk of bias due to lack of blinding of participants and allocation concealment (Li et al., 2023; Zheng et al., 2023). The meta-analysis showed that compared to the propofol group, the effect of the esketamine combination group on induction time was not significant (WMD −6.01; 95%CI −13.05, 1.03; *p* = 0.09; I^2^ = 98%). TSA indicated that the Z-curve of the induction time did not reach the boundary. As shown in Figure 3B.

#### 3.4.2 Vital signs

##### 3.4.2.1 Mean heart rate

**FIGURE 4:**
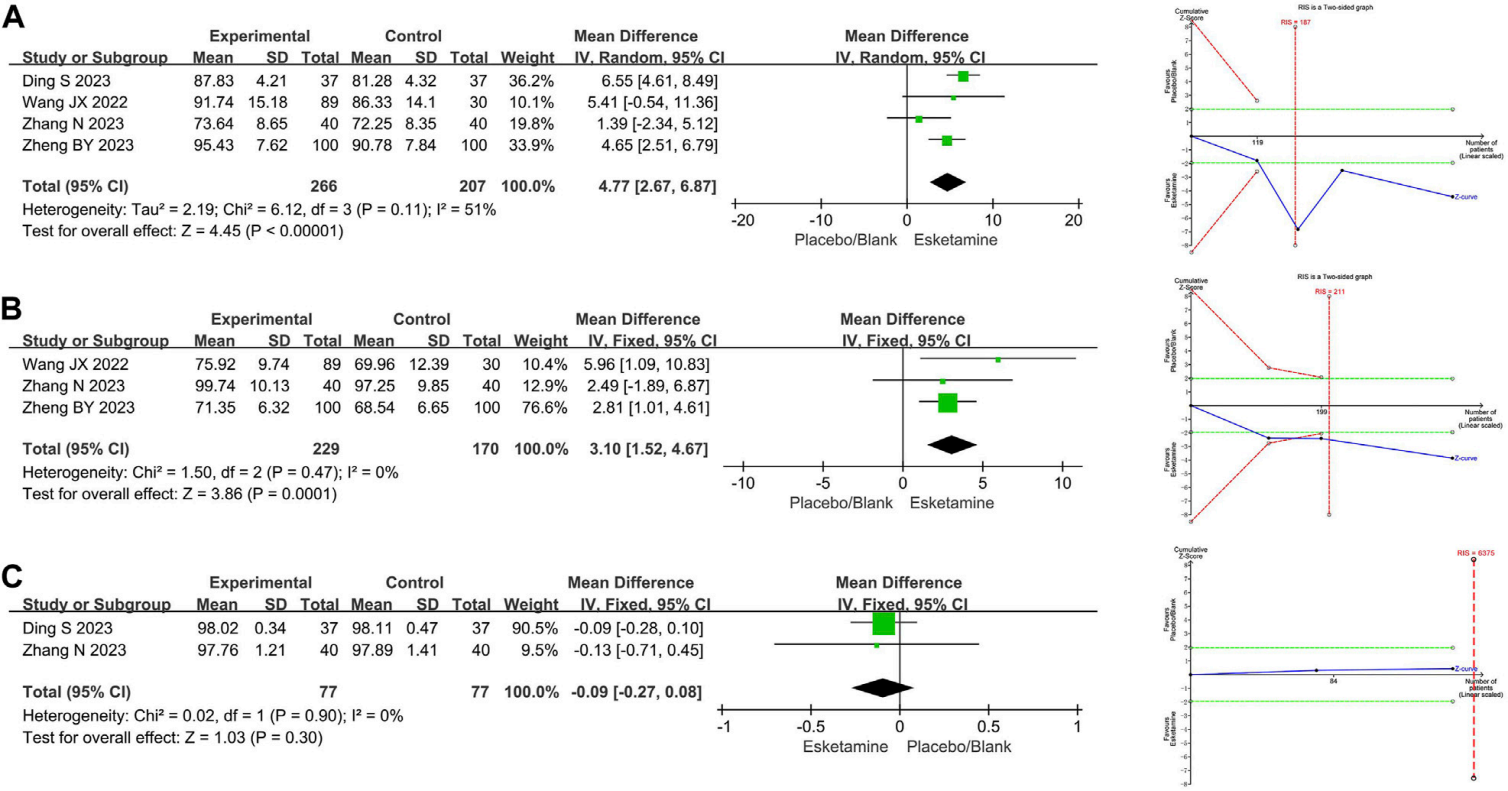
Meta-analysis and trial sequential analysis results for vital signs of esketamine in pediatric gastrointestinal endoscopy. **(A)** Mean heart rate; **(B)** Mean arterial pressure; **(C)** Mean blood oxygen. MD refers to the weighted mean difference.

Four RCTs compared mean heart rate between the esketamine combination group and the propofol group, and they included 473 children undergoing gastrointestinal endoscopy. Among them, three studies had a low risk of bias (Wang et al., 2022; Zhang et al., 2023; Ding et al., 2023), and one study had a high risk of bias due to lack of blinding of participants and allocation concealment (Zheng et al., 2023). The meta-analysis showed that compared to the propofol group, the esketamine combination group significantly increased the mean heart rate by 4.77 beats/min (WMD 4.77; 95% CI 2.67, 6.87; *p* < 0.00001; I^2^ = 51%). TSA indicated that the Z-curves of the mean heart rate crossed the boundary in the second study, suggesting it was conclusive. As shown in Figure 4A.

##### 3.4.2.2 Mean arterial pressure

Three RCTs compared mean arterial pressure between the esketamine combination group and the propofol group, and they included 399 children undergoing gastrointestinal endoscopy. Among them, two studies had a low risk of bias (Wang et al., 2022; Zhang et al., 2023), and one study had a high risk of bias due to lack of blinding of participants and allocation concealment (Zheng et al., 2023). The meta-analysis showed that compared to the propofol group, the esketamine combination group significantly increased the mean arterial pressure by 3.10 mmHg (WMD 3.10; 95% CI 1.52, 4.67; *p* = 0.0001; I^2^ = 0%). TSA indicated that the Z-curves of the mean arterial pressure crossed the boundary in the second study, suggesting it was conclusive. As shown in Figure 4B.

##### 3.4.2.3 Mean blood oxygen

Two RCTs compared mean blood oxygen between the esketamine combination group and the propofol group, and they included 154 children undergoing gastrointestinal endoscopy. Among them, two studies had low risk of bias (Zhang et al., 2023; Ding et al., 2023). The meta-analysis showed that compared to the propofol group, the effect of the esketamine combination group on mean blood oxygen was not significant (WMD −0.09; 95% CI −0.27, 0.08; *p* = 0.30; I^2^ = 0%). TSA indicated that the Z-curves of the mean blood oxygen did not reach the boundary. As shown in Figure 4C.

#### 3.4.3 Propofol consumption

**FIGURE 5:**
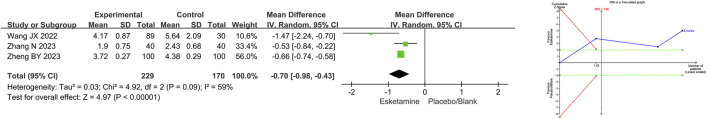
Meta-analysis and trial sequential analysis results for propofol consumption of esketamine in pediatric gastrointestinal endoscopy. MD refers to the weighted mean difference.

Three RCTs compared propofol consumption between the esketamine combination group and the propofol group, and they included 399 children undergoing gastrointestinal endoscopy. Among them, two studies had a low risk of bias (Wang et al., 2022; Zhang et al., 2023), and one study had a high risk of bias due to lack of blinding of participants and allocation concealment (Zheng et al., 2023). The meta-analysis showed that compared to the propofol group, the esketamine combination group significantly reduced the propofol consumption by 0.70 mg/kg (WMD −0.70; 95% CI −0.98, −0.43; *p* < 0.00001; I^2^ = 59%). TSA indicated that the Z-curve of propofol consumption crossed the boundary in the first study, suggesting that it was conclusive. As shown in Figure 5.

#### 3.4.4 Safety endpoint

##### 3.4.4.1 Total adverse events

Four RCTs compared total adverse events between the esketamine combination group and the propofol group, and they included 469 children undergoing gastrointestinal endoscopy. Among them, two studies had a low risk of bias (Wang et al., 2022; Ding et al., 2023), and two studies had a high risk of bias due to lack of blinding of participants and allocation concealment (Li et al., 2023; Zheng et al., 2023). The meta-analysis showed that compared to the propofol group, the effect of the esketamine combination group on total adverse events was not significant (RR 0.95; 95% CI 0.47, 1.95; *p* = 0.90; I^2^ = 70%). TSA indicated that the result of total adverse events was not conclusive. As shown in Table 2.

**TABLE 2:** Meta-analysis and trial sequential analysis results for the safety endpoints of esketamine in pediatric gastrointestinal endoscopy.

Outcome	Number of studies	Experimental (events/total)	Control (events/total)	I^2^/%	RR (95% CI)	*p*-value	TSA
Total adverse events	4	80/264	41/205	70	0.95 (0.47, 1.95)	0.90	No
Involuntary movement	4	11/215	27/215	0	0.41 (0.22, 0.76)	0.005	Yes
Choking	4	19/267	23/208	0	0.49 (0.26, 0.92)	0.03	No
Respiratory depression	4	3/215	8/215	0	0.41 (0.12, 1.41)	0.16	No
Vomiting	3	8/164	4/105	50	1.18 (0.17, 8.33)	0.87	No
Dizziness	2	51/127	10/68	0	1.98 (1.11, 3.56)	0.02	Yes

RR refers to risk ratio; TSA refers to trial sequential analysis.

##### 3.4.4.2 Involuntary movements

Four RCTs compared involuntary movements between the esketamine combination group and the propofol group, and they included 430 children undergoing gastrointestinal endoscopy. Among them, two studies had a low risk of bias (Zhang et al., 2023; Ding et al., 2023), and two studies had a high risk of bias due to lack of blinding of participants and allocation concealment (Li et al., 2023; Zheng et al., 2023). The meta-analysis showed that compared to the propofol group, the esketamine combination group significantly reduced the involuntary movements by 59% (RR 0.41; 95% CI 0.22, 0.76; *p* = 0.005; I^2^ = 0%). TSA indicated that the result of involuntary movements was conclusive. As shown in Table 2.

##### 3.4.4.3 Choking

Four RCTs compared choking between the esketamine combination group and the propofol group, and they included 475 children undergoing gastrointestinal endoscopy. Among them, two studies had a low risk of bias (Wang et al., 2022; Zhang et al., 2023), and two studies had a high risk of bias due to lack of blinding of participants and allocation concealment (Li et al., 2023; Zheng et al., 2023). The meta-analysis showed that compared to the propofol group, the esketamine combination group significantly reduced choking by 51% (RR 0.49; 95% CI 0.26, 0.92; *p* = 0.03; I^2^ = 0%). TSA indicated that the result of choking was not conclusive. As shown in Table 2.

##### 3.4.4.4 Respiratory depression

Four RCTs compared respiratory depression between the esketamine combination group and the propofol group, and they included 430 children undergoing gastrointestinal endoscopy. Among them, two studies had a low risk of bias (Zhang et al., 2023; Ding et al., 2023), and two studies had a high risk of bias due to lack of blinding of participants and allocation concealment (Li et al., 2023; Zheng et al., 2023). The meta-analysis showed that compared to the propofol group, the effect of the esketamine combination group on respiratory depression was not significant (RR 0.41; 95% CI 0.12, 1.41; *p* = 0.16; I^2^ = 0%). TSA indicated that the result of respiratory depression was not conclusive. As shown in Table 2.

##### 3.4.4.5 Vomiting

Three RCTs compared vomiting between the esketamine combination group and the propofol group, and they included 269 children undergoing gastrointestinal endoscopy. Among them, two studies had a low risk of bias (Wang et al., 2022; Ding et al., 2023), and one study had a high risk of bias due to lack of blinding of participants and allocation concealment (Li et al., 2023). The meta-analysis showed that compared to the propofol group, the effect of the esketamine combination group on vomiting was not significant (RR 1.18; 95% CI 0.17, 8.33; *p* = 0.87; I^2^ = 50%). TSA indicated that the result of vomiting was not conclusive. As shown in Table 2.

##### 3.4.4.6 Dizziness

Two RCTs compared dizziness between the esketamine combination group and the propofol group, and they included 195 children undergoing gastrointestinal endoscopy. Among them, one study had a low risk of bias (Wang et al., 2022), and one study had a high risk of bias due to lack of blinding of participants and allocation concealment (Li et al., 2023). The meta-analysis showed that compared to the propofol group, the esketamine combination group significantly increased dizziness by 98% (RR 1.98; 95% CI 1.11, 3.56; *p* = 0.02; I^2^ = 0%). TSA indicated that the result of dizziness was conclusive. As shown in Table 2.

### 3.5 Subgroup analysis

Since only five studies were included in this meta-analysis and the reporting of participant characteristics was incomplete, it was difficult to perform subgroup analysis based on participant characteristics. Thus, we solely conducted a subgroup analysis based on the dose of esketamine to assess the impact of clinical heterogeneity on outcomes, as depicted in Table 3. Among them, esketamine doses of ≤0.3 mg/kg were designated as low-dose (Yang et al., 2023), whereas doses exceeding 0.3 mg/kg were considered high-dose.

**TABLE 3:** The results of subgroup analysis based on the dose of esketamine.

Outcome	Subgroup	Number of studies	I^2^/%	WMD/RR (95% CI)	*p*-value
Recovery time	Low-dose	4	88	−2.05 (−3.81, −0.29)	0.02
	High-dose	2	85	0.50 (−8.54, 9.55)	0.91
Induction time	Low-dose	2	99	−8.16 (−16.70, 0.37)	0.06
	High-dose	1	0	−1.20 (−5.52, 3.12)	0.59
Mean heart rate	Low-dose	3	66	4.71 (0.87, 8.55)	0.02
	High-dose	2	0	4.67 (2.64, 6.70)	<0.00001
Mean artery pressure	Low-dose	2	58	5.04 (−0.72, 10.81)	0.09
	High-dose	2	0	3.04 (1.35, 4.73)	0.0004
Mean blood oxygen	Low-dose	2	0	−0.09 (−0.27, 0.08)	0.30
	High-dose	0	-	—	—
Propofol consumption	Low-dose	2	63	−0.80 (−1.49, −0.11)	0.02
	High-dose	2	81	−1.03 (−1.92, −0.15)	0.02
Total adverse events	Low-dose	3	34	1.27 (0.76, 2.11)	0.37
	High-dose	2	68	0.63 (0.21, 1.89)	0.41
Involuntary movement	Low-dose	3	0	0.42 (0.22, 0.80)	0.008
	High-dose	1	0	0.33 (0.04, 3.15)	0.34
Choking	Low-dose	3	5	0.53 (0.26, 1.08)	0.08
	High-dose	2	0	0.59 (0.24, 1.48)	0.26
Respiratory depression	Low-dose	3	0	0.38 (0.09, 1.62)	0.19
	High-dose	1	0	0.50 (0.05, 5.43)	0.57
Vomiting	Low-dose	3	45	1.61 (0.54, 4.82)	0.40
	High-dose	1	0	0.50 (0.03, 7.72)	0.62
Dizziness	Low-dose	2	0	1.64 (0.83, 3.23)	0.15
	High-dose	1	0	2.19 (1.16, 4.11)	0.02

WMD refers to weighted mean difference; RR refers to risk ratio.

Subgroup analysis revealed that low-dose esketamine reduced recovery time (WMD −2.05; 95% CI −3.81, −0.29; *p* = 0.02; I^2^ = 88%), propofol consumption (WMD −0.80; 95% CI −1.49, −0.11; *p* = 0.02; I^2^ = 63%), and involuntary movement (RR 0.42; 95% CI 0.22, 0.80; *p* = 0.008; I^2^ = 0%), and increased mean heart rate (WMD 4.71; 95% CI 0.87, 8.55; *p* = 0.02; I^2^ = 66%), with no significant effects on other outcomes. High-dose esketamine reduced propofol consumption (WMD −1.03; 95% CI −1.92, −0.15; *p* = 0.02; I^2^ = 81%) and increased mean heart rate (WMD 4.67; 95% CI 2.64, 6.70; *p* < 0.00001; I^2^ = 0%), mean artery pressure (WMD 3.04; 95% CI 1.35, 4.73; *p* = 0.0004; I^2^ = 0%), and dizziness (RR 2.19; 95% CI 1.16, 4.11; *p* = 0.02; I^2^ = 0%), with no significant effect on other outcomes. However, in this subgroup analysis, heterogeneity in recovery time, induction time, mean heart rate, propofol consumption, total adverse events, and vomiting remained significant, suggesting that their heterogeneity was not due to clinical differences in the esketamine dose.

### 3.6 Sensitivity analysis

#### 3.6.1 Sensitivity analysis based on blinding of participants

We performed sensitivity analyses for studies in which blinding of participants was low-risk, which was to assess the impact of methodological heterogeneity on the results, as shown in Table 4. The sensitivity analysis based on blinding of participants revealed a change in the significance of esketamine on induction time (WMD −12.51; 95% CI −13.82, −11.20; *p* < 0.00001; I^2^ = 0%) and choking (WMD 0.54; 95% CI 0.27, 1.07; *p* = 0.08; I^2^ = 45%), suggesting that the results reported by the meta-analysis for induction time and choking were not robust. The significance of the remaining outcomes reported by the sensitivity analysis and meta-analysis is similar, suggesting that their results were robust. In addition, in this sensitivity analysis, the heterogeneity of recovery time, induction time and vomiting was significantly reduced, indicating that the heterogeneity observed in the meta-analysis was related to the absence of blinding of participants.

**TABLE 4:** The results of sensitivity analysis based on blinding of participants.

Outcome	Number of studies	I^2^/%	WMD/RR (95% CI)	*p*-value
Recovery time	3	27	−1.30 (−1.83, −0.77)	<0.00001
Induction time	1	0	−12.51 (−13.82, −11.20)	<0.00001
Mean heart rate	3	65	4.59 (1.02, 8.15)	0.01
Mean artery pressure	2	7	4.04 (0.78, 7.30)	0.02
Mean blood oxygen	2	0	−0.09 (−0.27, 0.08)	0.30
Propofol consumption	2	80	−0.93 (−1.84, −0.02)	0.045
Total adverse events	2	73	1.20 (0.40, 3.56)	0.75
Involuntary movement	2	0	0.43 (0.22, 0.84)	0.01
Choking	2	45	0.54 (0.27, 1.07)	0.08
Respiratory depression	2	0	0.67 (0.11, 3.88)	0.65
Vomiting	2	0	0.56 (0.13, 2.37)	0.43
Dizziness	1	0	1.98 (1.06, 3.70)	0.03

WMD refers to weighted mean difference; RR refers to risk ratio.

#### 3.6.2 Sensitivity analysis based on leave-one-out method

To further explore the sources of heterogeneity in mean heart rate, propofol consumption, and total adverse events, we employed leave-one-out sensitivity analysis. Firstly, the leave-one-out sensitivity analysis revealed that the heterogeneity of mean heart rate originated from the study of Zhang N et al. The sensitivity analysis after deleting this study demonstrated consistent results with the meta-analysis (WMD 5.68; 95% CI 4.28, 7.08; *p* < 0.00001; I^2^ = 0%), suggesting that the meta-analysis result of mean heart rate was robust. By reviewing the included studies on mean heart rate, we speculate that the heterogeneity may be related to the high ASA I rate of 87.5% and a dose of only 0.2 mg/kg of esketamine in Zhang N et al.

Secondly, the heterogeneity of propofol consumption originated from the study by Wang JX et al. The sensitivity analysis after deleting this study showed consistent results with the meta-analysis (WMD −0.65; 95% CI −0.73, −0.58; *p* < 0.00001; I^2^ = 0%), suggesting that the meta-analysis result of propofol consumption was robust. By reviewing the included studies on propofol consumption, we speculate that the heterogeneity may be associated with the inclusion of esketamine at dose as high as 0.7 mg/kg by Wang JX et al.

Thirdly, the heterogeneity of total adverse events mainly came from the study by Zheng BY et al. The sensitivity analysis after deleting this study demonstrated consistent results with the meta-analysis (RR 1.41; 95% CI 0.97, 2.04; *p* = 0.07; I^2^ = 48%), suggesting that the meta-analysis result of total adverse events was robust. However, when reviewing the included studies on total adverse events, we did not find obvious methodological and clinical heterogeneity between the study by Zheng BY et al. and others. And there was still moderate heterogeneity after removing the study by Zheng BY et al. Therefore, we attribute the heterogeneity of total adverse events to statistical heterogeneity.

### 3.7 Publication bias

**FIGURE 6:**
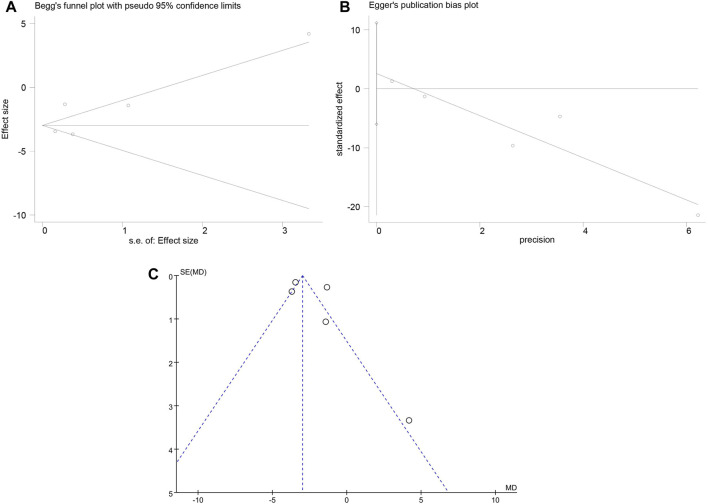
Publication bias results. **(A)** Begg’s test; **(B)** Egger’s test; **(C)** Funnel plot.

Recovery time was defined as the primary efficacy endpoint. The Begg’s test for recovery time showed a *p*-value of 0.327 and the Egger’s test for recovery time showed a *p*-value of 0.413, suggesting no significant publication bias. However, the funnel plot for recovery time showed asymmetric scatter distribution on both sides, indicating potential publication bias. As shown in Figure 6.

## 4 Discussion

### 4.1 Background and significance

Gastrointestinal endoscopy remains the primary means for diagnosing gastrointestinal diseases (Wallace et al., 2017). With advancements in anesthesia techniques, painless gastrointestinal endoscopy is gradually replacing conventional methods (Li et al., 2023). Compared to conventional gastrointestinal endoscopy, painless gastrointestinal endoscopy not only alleviates patient discomfort but also improves the quality and efficiency of examinations, making it more widely accepted by physicians and patients (Zhan et al., 2022). Propofol is one of the most commonly used drugs for painless gastrointestinal endoscopy (Zhang et al., 2020). Although complete sedation can be achieved with the clinical dose of propofol alone, its potential for respiratory and cardiovascular depression still concerns clinicians (Wadhwa et al., 2017; Sneyd et al., 2022). Studies have shown that drugs such as lidocaine, ketamine and esketamine can assist in anesthesia during gastrointestinal endoscopy, and they are able to reduce propofol consumption and related adverse events during sedation (Türk et al., 2014; Forster et al., 2018; Liu et al., 2023). A recent meta-analysis demonstrated that esketamine effectively reduces recovery time, propofol consumption, and related complications in adults undergoing gastrointestinal endoscopy (Lian et al., 2023). However, due to limited clinical evidence, it remains unclear whether the same benefits exist in children. To our knowledge, this is the first meta-analysis and TSA evaluating the role of esketamine in the pediatric gastrointestinal endoscopy, aiming to provide higher-quality evidence for the use of esketamine in specific populations.

### 4.2 Pharmacological description

Esketamine is a non-competitive inhibitor of NMDA receptors, exerting anesthetic and analgesic effects by blocking NMDA receptor (Wallace et al., 2017). Esketamine is both water-soluble and fat-soluble with high bioavailability, which is mainly administered orally, nasally and intravenously (Cohen et al., 2006; Xu et al., 2023). Following intravenous administration, esketamine reaches peak blood concentration within 1–2 min, exhibiting widespread distribution throughout the body and rapid crossing of the blood-brain barrier (Riphaus et al., 2013; Zhou et al., 2021). It has a mean half-life of 7–12 h and is metabolized in the organism by hepatic microsomal enzymes to form S-desketamine (Zheng et al., 2018), which is excreted by glucuronidation (FDA-approved drug: Spravato esketamine nasal spray, 2024). The pharmacological effects of esketamine mainly involve the following aspects: Firstly, esketamine mediates excitatory glutamate neurons by directly inhibiting NMDA receptors, producing potent analgesia, loss of consciousness, and antidepressant effects (Hope et al., 2023). Secondly, esketamine exerts antidepressant effects by regulating the levels of neurotransmitters such as γ-aminobutyric acid, dopamine, and serotonin as well as enhancing synaptic plasticity (Vollenweider et al., 2000; du Jardin et al., 2016; Jelen et al., 2021). Thirdly, esketamine indirectly stimulates the cardiovascular system by promoting catecholamine release, inhibiting norepinephrine reuptake, and activating the sympathetic nervous system to produce sympathomimetic effects, which in turn produces elevated blood pressure and accelerated heart rate (Kiriyama et al., 2014). Lastly, esketamine antagonizes histamine and enhances norepinephrine to alleviate bronchial smooth muscle spasms, thus improving pulmonary compliance (Rutman, 2009).

### 4.3 Efficacy analysis

In terms of propofol consumption, this study reveals that esketamine significantly reduced the propofol consumption by 0.70 mg/kg, indicating its ability to alleviate pediatric anesthesia burden. This is similar to the results of the meta-analysis reported by Lian et al. (2023), who found that esketamine effectively reduced propofol consumption by 1.68 mg/kg in adults undergoing gastrointestinal endoscopy. This evidence in propofol consumption may be related to the antagonistic effect of esketamine on NMDA receptors. By blocking the activation of NMDA receptors, esketamine inhibits the excitatory neurotransmission, thus providing sedative and analgesic effects (Zanos et al., 2018). Meanwhile, esketamine also reduces NO production by blocking NMDA receptors, thereby attenuating the inhibitory effect of NO on gamma-aminobutyric acid (GABA) (Luo and Cizkova, 2000). And this contributes to the sedative effects of propofol, as GABA-mediated central nervous system inhibition is its primary mechanism of sedation (Ito et al., 1999). Therefore, esketamine exerts sedative and analgesic effects by directly inhibiting NMDA receptors and indirectly activating GABA receptors, thereby reducing the need for propofol during gastrointestinal endoscopy.

In terms of vital signs, this study shows that esketamine significantly increased mean heart rate by 4.77 beats/min and mean arterial pressure by 3.10 mmHg, suggesting that it is ability to attenuate the pediatric cardiovascular inhibition caused by propofol. The meta-analysis by Lian et al. (2023) did not report the effect of esketamine on mean heart rate and mean arterial pressure, but they found that esketamine significantly reduced bradycardia by 29% and hypotension by 69% in adults undergoing gastrointestinal endoscopy. The positive effects of esketamine on the cardiovascular system may be related to the following mechanisms: First, the propofol consumption required to maintain anesthesia was reduced by approximately 0.70 mg/kg with esketamine participation. Given that propofol increases the risk of hypotension and bradycardia in a dose-dependent manner (Claeys et al., 1988), decreasing propofol consumption can reduce its cardiovascular inhibitory effects. Second, esketamine promotes the release of norepinephrine through a negative feedback mechanism and inhibits the reuptake of norepinephrine by neurons, thereby exerting sympathomimetic effects and promoting an increase in heart rate and blood pressure (Kohrs and Durieux, 1998). Third, esketamine reduces cardiac parasympathetic activity by blocking brainstem parasympathetic nerves, thereby increasing heart rate and blood pressure (Irnaten et al., 2002). Therefore, in addition to reducing propofol consumption, the sympathomimetic and antiparasympathetic effects of esketamine itself counteract the cardiovascular depression of propofol, thereby stabilizing children’s vital signs.

In terms of time endpoints, this study shows that esketamine significantly reduced the recovery time by 2.34 min, suggesting that it contributes to postoperative recovery of consciousness in children. Our results are supported by a meta-analysis by Lian et al. (2023), who reported that esketamine effectively reduced the recovery time by 0.96 min in adult gastrointestinal endoscopy. This effect is attributed to the NMDA-modulating properties of esketamine. It forms a synergistic effect with propofol by non-competitively inhibiting NMDA receptors (Feng et al., 2022), thereby reducing the propofol consumption. Since the plasma concentration of propofol is positively correlated with its dose, reducing the dose of propofol will weaken its sedative effect, thereby prompting children to wake up faster (Zhang et al., 2021). It is worth noting that although the induction time in the meta-analysis report was not statistically significant, sensitivity analysis based on blinding of participants found that esketamine significantly reduced the induction time. Therefore, the meta-analysis result of induction time are not robust, and more research is needed in the future to explore the impact of esketamine on induction time in pediatric gastrointestinal endoscopy.

The included studies also reported some other benefits of esketamine in gastrointestinal endoscopy in children. They found that esketamine significantly improves the success rate of first-dose anesthesia (Ding et al., 2023), the success rate of the first endoscope insertion (Wang et al., 2022), the satisfaction of endoscopists (Wang et al., 2022) and the satisfaction of patients’ families (Zheng et al., 2023). It suggests that the anesthesia regimen of esketamine in combination with propofol improves the success rate of pediatric gastrointestinal endoscopy and gains more support from physicians and children’s families. The included studies also showed that esketamine significantly reduces injection pain (Zhang et al., 2023), FLACC scores (Ding et al., 2023), PAED scores (Ding et al., 2023) and Ambesh scores (Zhang et al., 2023). This implies that esketamine is effective in reducing pain and agitation in children during gastrointestinal endoscopy and enhancing their tolerance to gastrointestinal endoscopy. This evidence supports that esketamine achieves additional benefits in assisting propofol anesthesia.

### 4.4 Safety analysis

In terms of safety endpoints, this study demonstrates no significant effect of esketamine on total adverse events, indicating that esketamine has a favorable overall safety profile. On individual adverse events, esketamine significantly reduced involuntary movements by 59% and choking by 51% compared to the control group. Although the sensitivity analysis based on blinding of participants did not find a benefit of choking, we speculate that this non-significant result may be attributed to an insufficient sample size. A meta-analysis by Lian et al. (2023) supported the benefit of esketamine in reducing involuntary movements. They found that esketamine reduces involuntary movements by 24% but did not analyze the effect of esketamine on choking. The potential benefits of esketamine in reducing involuntary movements and choking may stem from its stronger central nervous system inhibition. Esketamine enhances the central nervous system inhibition of propofol by blocking NMDA receptors, resulting in stronger sedation and analgesia (Zanos et al., 2018). This synergistic effect helps to reduce peripheral and central nociception and enhance pain inhibition (Kang et al., 2021), thereby reducing involuntary movements and choking induced by gastrointestinal stimulation.

The results of this study also demonstrates that the incidence of respiratory depression and vomiting is comparable between the esketamine combination group and the control group. A meta-analysis by Lian et al. (2023) supported that esketamine do not have a significant effect on the risk of vomiting in adults, but it pointed to a 67% reduction in the risk of respiratory depression in adults with esketamine (Lian et al., 2023). Another clinical trial in the Netherlands also confirmed that subanesthetic doses of esketamine have a stimulatory effect on the respiratory center (Jonkman et al., 2018). This difference may be related to the propofol consumption in children. Since the propofol consumption in children is lower than in adults, it may not be sufficient to highlight the respiratory depression of propofol. This may result in the failure of esketamine to improve the respiratory depression of propofol.

It is worth noting that esketamine is not an absolute benefit factor for gastrointestinal endoscopy in children. This study found that esketamine almost doubled the risk of dizziness (RR 1.98, 95% CI 1.11∼3.56, *p* = 0.02), which may be another manifestation of central nervous system inhibition caused by its NMDA blocking effects. However, a meta-analysis by Lian et al. (2023) showed that esketamine is not associated with the risk of dizziness in adults. We speculate that this difference is due to the insufficient tolerance of children to esketamine. Therefore, anesthesiologists need to be alert to the occurrence of dizziness when esketamine is used for gastrointestinal endoscopy in children. Considering that the adverse events of esketamine are closely related to the dose, dizziness may be associated with higher doses of esketamine. Therefore, we conducted a supplementary analysis on low-dose (≤0.3 mg/kg) esketamine for gastrointestinal endoscopy in children.

In fact, previous studies support the use of esketamine for a number of other pediatric procedures. A clinical study encompassing 100 children revealed shorter recovery times, smoother hemodynamics, and fewer adverse events in endoscopic adenoid tonsillectomy in the esketamine combined with dexmedetomidine compared with the dexmedetomidine (Li et al., 2022). Furthermore, esketamine has also been reported to significantly reduce the incidence of emergent agitation and inflammation levels after tonsillectomy (Li et al., 2022; Liu et al., 2023). Another study involving 77 children undergoing hypospadias surgery demonstrated that, compared with hydromorphone combined with sacral block, esketamine combined with sacral block significantly reduced the incidence of hypotension and respiratory depression as well as shortened the time to first bowel movement (Xu et al., 2023). Additionally, Zhang et al., (2023b) conducted a meta-analysis of 19 clinical trials and pointed out that, compared with the placebo or blank, the esketamine significantly shortened the postoperative recovery time, reduced pain and complication rates, and improved quality of life in pediatric patients. These pieces of evidence suggest that esketamine is a worthwhile pediatric anesthesia adjuvant, indirectly supporting our findings.

### 4.5 Low-dose effects analysis

This analysis indicates that low-dose esketamine still significantly reduces recovery time, propofol consumption, involuntary movements, and increases mean heart rate, and that these benefits are conclusive. Although low-dose esketamine lost the benefits of increased mean arterial pressure and reduced choking, it no longer carry an increased risk of dizziness. This may be due to the relatively weak central nervous system inhibition mediated by low-dose esketamine, thereby leading to a reduction in its effects on reducing choking and inducing dizziness. Meanwhile, considering the relatively weak sympathomimetic and antiparasympathomimetic effects of low-dose esketamine, it may have contributed to the less pronounced effect in increasing mean arterial pressure. In fact, safety is more important than efficacy in pediatric gastrointestinal endoscopy (Su et al., 2023). When carrying out gastrointestinal endoscopy, it is necessary to choose the anesthetic regimen that poses the lowest potential risk to the children. Although low-dose esketamine does not reduce choking and increase mean arterial pressure, it helps children avoid additional risks of dizziness. Therefore, we recommend that anesthesiologists choose an anesthetic strategy of low-dose esketamine combined with propofol for gastrointestinal endoscopy in children.

### 4.6 New knowledge and clinical value

This study revealed that esketamine, as an adjunct to propofol anesthesia, significantly reduced the propofol consumption, propofol-induced cardiovascular depression, recovery time, and adverse events such as involuntary movements and choking in pediatric gastrointestinal endoscopy, but increased the risk of dizziness. Unlike conventional doses, low-dose (≤0.3 mg/kg) esketamine achieved benefits without increasing the risk of dizziness in pediatric gastrointestinal endoscopy. This new knowledge demonstrates that low-dose esketamine is a safe and effective adjunct to propofol anesthesia and has potential for use in pediatric gastrointestinal endoscopy. We recommend that clinicians and anesthesiologists take low-dose esketamine into consideration during pediatric gastrointestinal endoscopy and construct an anesthesia plan for low-dose esketamine combined with propofol. This approach will aid in enhancing the stability and safety of pediatric gastrointestinal endoscopy anesthesia, benefiting a larger number of children.

### 4.7 Limitations and perspectives

It is undeniable that this study has been influenced and limited by some factors. Firstly, only five clinical trials and 549 samples were included in this study, which may lead to a decrease in the precision of the meta-analysis results. In addition, insufficient sample size may make small differences difficult to detect, and the insignificant differences in respiratory depression may be due to insufficient sample size. Secondly, TSA revealed that the benefit of esketamine in reducing choking incidence was inconclusive, and further clinical trials are needed to investigate the impact of esketamine on choking. Thirdly, four included studies did not mention allocation concealment, and two studies did not mention intervention blinding of participants, which increased the potential risk of selection bias and implementation bias. It is often the cause of potential methodological heterogeneity. Fourthly, there are some differences in average age, male ratio, and ASA I ratio among the included studies, which may lead to potential clinical heterogeneity. However, due to the limited number of studies included, we are unable to conduct subgroup analysis based on these factors. Fifthly, all of the trial centers included in the study were located in China, which means that the study mainly reveals the effects of esketamine on Chinese children. Due to the fact that the FDA has not yet approved esketamine for anesthesia and clinical trials have not yet been conducted in other countries, the role of esketamine in children of different races is unclear. Sixthly, although this study suggests that low-dose esketamine combined with propofol is a safe and effective anesthesia strategy, it is still not clear what the difference in efficacy is between different doses of esketamine in the range of 0.3 mg/kg.

Future research can be improved in the following aspects: First, establish research centers in other countries to explore the impact of esketamine on pediatric gastrointestinal endoscopy of different races. Second, high-quality clinical trials continue to be conducted to explore esketamine’s effect on different outcomes of pediatric gastrointestinal endoscopy, providing additional evidence for evidence-based research. Third, clinical trials can be designed to compare the benefits and risks of different low-dose esketamine in pediatric gastrointestinal endoscopy and explore the optimal dose of esketamine to assist propofol anesthesia.

## 5 Conclusion

Esketamine is an effective adjuvant anesthesia for children undergoing gastrointestinal endoscopy, but the potential risk of dizziness should be noted. Low-dose (≤0.3 mg/kg) esketamine does not increase the risk of dizziness, which is a safe and effective adjuvant anesthesia. However, the findings is not confirmative due to small number of the included studies, and more similar clinical studies are needed in the future to validate this discovery.

## Data Availability

The original contributions presented in the study are included in the article/supplementary material, further inquiries can be directed to the corresponding authors.

## Abbreviations

NMDA, N-methyl-D-aspartate; EMA, European Medicines Agency; NMPA, National Medical Products Administration; FDA, Food and Drug Administration; TSA, Trial sequential analysis; GABA, Gamma-aminobutyric acid.
